# Statins as Potential Chemoprevention or Therapeutic Agents in Cancer: a Model for Evaluating Repurposed Drugs

**DOI:** 10.1007/s11912-021-01023-z

**Published:** 2021-02-13

**Authors:** Nalinie Joharatnam-Hogan, Leo Alexandre, James Yarmolinsky, Blossom Lake, Nigel Capps, Richard M Martin, Alistair Ring, Fay Cafferty, Ruth E Langley

**Affiliations:** 1 MRC Clinical Trials Unit at University College London, 90 High Holborn, London, WC1V 6LJ UK; 2 Norfolk and Norwich University Hospital NHS Trust, Norwich, UK; 3 Norwich Medical School, University of East Anglia, Norwich, UK; 4 MRC Integrative Epidemiology Unit, University of Bristol, Bristol, UK; 5 The Shrewsbury and Telford Hospital NHS Trust, Shrewsbury, UK; 6 Medical Research Council (MRC) Integrative Epidemiology Unit; Population Health Sciences, Bristol Medical School, University of Bristol, Bristol, UK; 7 National Institute for Health Research Bristol Biomedical Research Centre, Bristol, UK; 8 Royal Marsden Hospital NHS Foundation Trust, Sutton, UK

**Keywords:** Drug repurposing, Statins, Cancer, Drug development

## Abstract

**Purpose of Review:**

Repurposing established medicines for a new therapeutic indication potentially has important global and societal impact. The high costs and slow pace of new drug development have increased interest in more cost-effective repurposed drugs, particularly in the cancer arena. The conventional drug development pathway and evidence framework are not designed for drug repurposing and there is currently no consensus on establishing the evidence base before embarking on a large, resource intensive, potential practice changing phase III randomised controlled trial (RCT). Numerous observational studies have suggested a potential role for statins as a repurposed drug for cancer chemoprevention and therapy, and we review the strength of the cumulative evidence here.

**Recent Findings:**

In the setting of cancer, a potential repurposed drug, like statins, typically goes through a cyclical history, with initial use for several years in another disease setting, prior to epidemiological research identifying a possible chemo-protective effect. However, further information is required, including review of RCT data in the initial disease setting with exploration of cancer outcomes. Additionally, more contemporary methods should be considered, such as Mendelian randomization and pharmaco-epidemiological research with “target” trial design emulation using electronic health records. Pre-clinical and traditional observational data potentially support the role of statins in the treatment of cancer; however, randomised trial evidence is not supportive. Evaluation of contemporary methods provides little added support for the use of statin therapy in cancer.

**Summary:**

We provide complementary evidence of alternative study designs to enable a robust critical appraisal from a number of sources of the go/no-go decision for a prospective phase III RCT of statins in the treatment of cancer.

## Introduction

From a global cancer perspective, repurposing established medicines for a new clinical indication is potentially an important strategy, particularly due to the increasing cancer burden in low- and middle-income countries where access to costly new therapies and technologies is limited. A major advantage of assessing repurposing possibilities is that many of the early steps in the drug development pathway, i.e., phase I/II trials establishing safety and tolerability, are already completed and generic formulations available. However, phase III randomised controlled trials (RCTs) are still needed to establish efficacy for the new clinical indication.

**Fig. 1:**
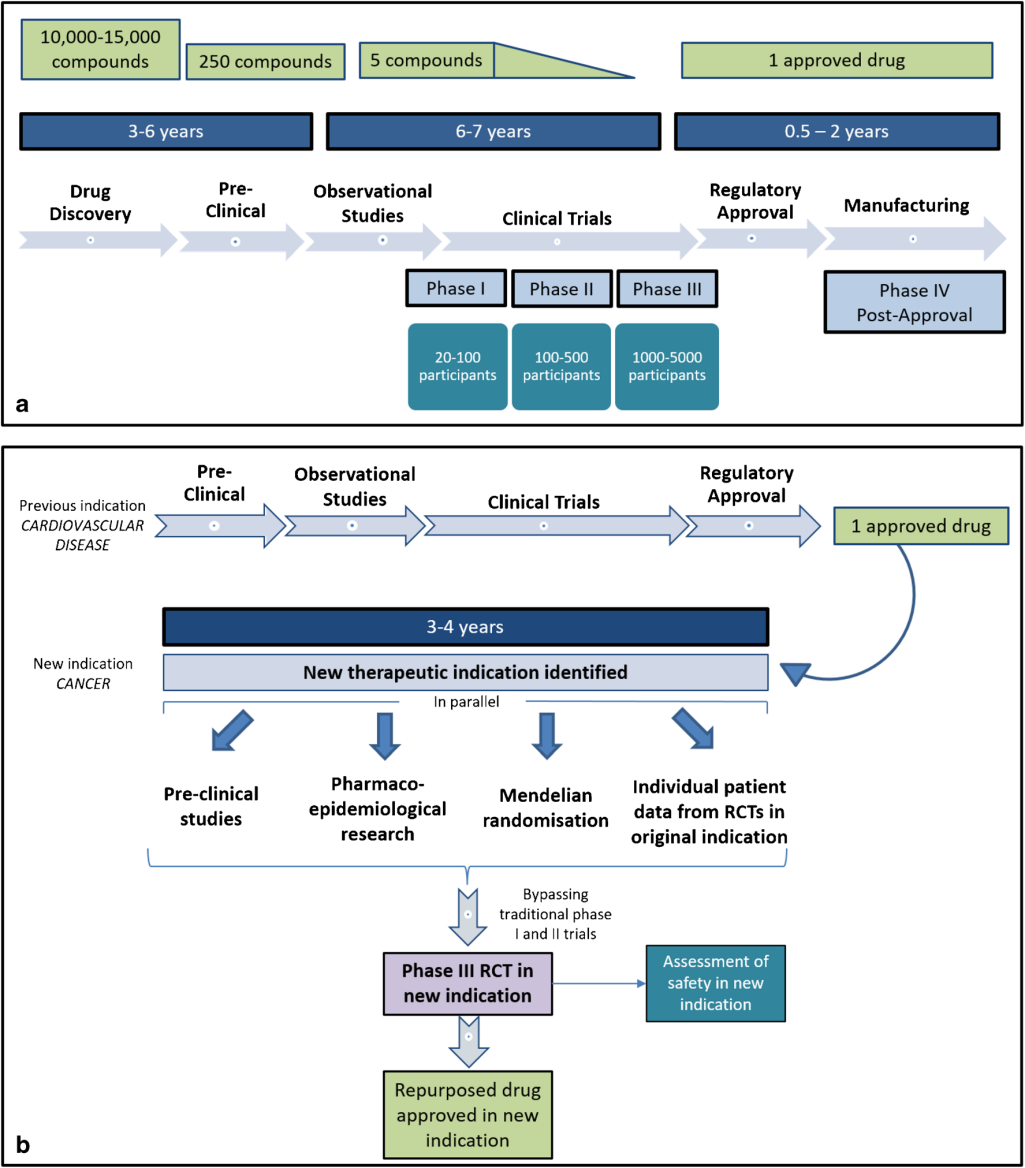
**a** Standard drug development pathway^97^. **b** Proposed drug development pathway for repurposed drugs

For new drugs, there is an established developmental pathway from pre-clinical evidence, through first in man studies, phase I and II trials leading to a phase III RCT (Fig. 1a). For repurposed drugs, the evidence base is different and there is no established consensus as to what this should comprise and how it should be appraised in order to prioritise potential repurposed drugs for phase III evaluation. Pre-clinical evidence can be complemented by epidemiological data, previous randomised trials, and Mendelian randomization or more modern genetic studies.

Some would argue that any low-cost, established drug with minimal toxicity and some evidence of anti-tumour activity is a candidate for phase III evaluation particularly in tumour types with poor outcomes. Proponents of such an approach argue there is little to lose, as long as the trial participants are fully informed. However, a phase III trial particularly in the adjuvant setting is a costly, resource intensive, and long-term undertaking and the scientific rationale underpinning the trial needs to be robustly evaluated.

**Fig. 2:**
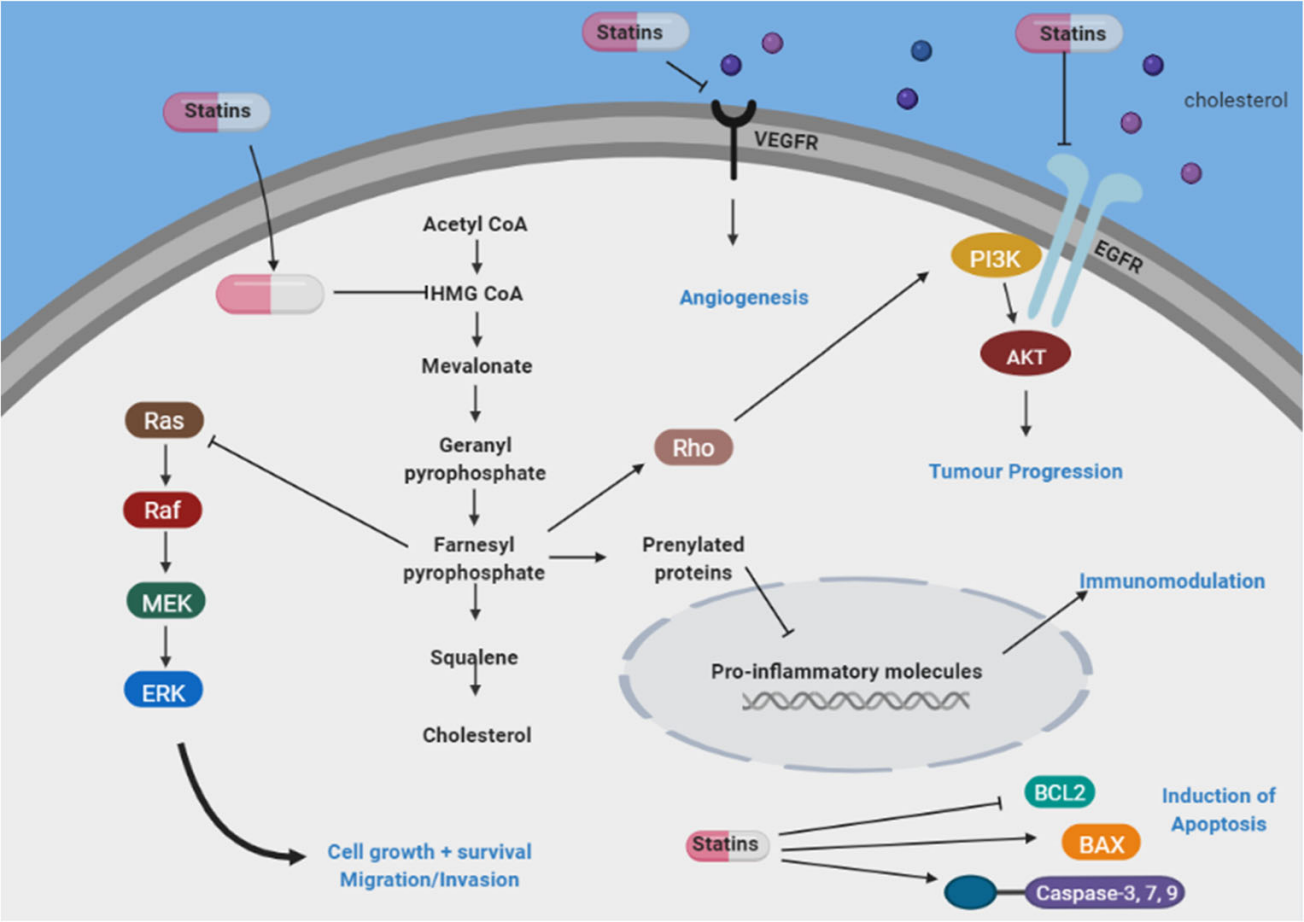
The mevalonate pathway and possible mechanism of action of statins

Statins, the most widely prescribed lipid-lowering agents, are inhibitors of the 3-hydroxy-3-methylglutaryl-coenzyme A reductase (HMGCR) enzyme, the rate-limiting step in the mevalonate pathway (Fig. 2). Numerous large RCTs in a range of populations have confirmed the role of statins for the prevention and treatment of cardiovascular and cerebrovascular disease, and they have become well-established therapy in this setting [1–3]. Beyond their lipid-lowering action and cardiovascular benefits, evidence has suggested statins may have multiple pleiotropic actions, including anti-inflammatory, antioxidant, anti-proliferative, and immunomodulatory effects, potentially yielding clinically relevant anticancer properties [4].

The existence of extensive randomised data evaluating statins for cardiovascular disease (CVD) provides an opportunity to interrogate these data for evidence of clinical effects on cancer outcomes. A similar approach was taken for aspirin. Meta-analyses of individual patient data from large RCTs of aspirin use in CVD prevention demonstrated significant reductions in cancer incidence, and risk of metastases [5]. These findings supported results from observational and pre-clinical data [6, 7]. Consequently, aspirin is now under investigation in several RCTs for the prevention of metastases [8].

Similarly, attention has turned to statins as a repurposed drug in the treatment of cancer. This review summarises the possible anticancer mechanism of action of statins and the breadth of evidence for and against its use in the prevention and treatment of cancer. Relative to previous reviews, a wider range of data sources and methods of evaluating the data is presented here, including the role of pharmaco-epidemiological research and Mendelian randomization to minimise potential confounding limiting the evaluation of observational studies [9]. We also present a novel model for assessing other drugs where there is potential for repurposing (Fig. 1b).

## Potential Anticancer Mechanisms of Action of Statins

Inhibition of HMGCR leads to a decrease in mevalonate and downstream cholesterol biosynthesis, as well as the inhibition of other isoprenoid metabolites, required for a number of cellular functions and post-translational modification of cell signalling proteins [10] (Fig. 2). Several in vitro studies have demonstrated that statins act to inhibit a number of the hallmarks of cancer, including inhibition of angiogenesis, induction of apoptosis, repression of tumour metastases, and inhibition of tumour cell growth [11] (Fig. 2). Statins vary in their solubility, with hydrophilic statins more hepato-selective and lipophilic statins achieving a higher level of exposure in non-hepatic tissue [12]. Based on this classification, lipophilic statins, including simvastatin and atorvastatin, are considered to exert more pleiotropic effects and are, therefore, potentially more effective as anticancer therapies.

In vivo experiments have proven more conflicting. Early studies in animal models raised concerns that statins may have carcinogenic properties [11]. In rodent studies, administration of fluvastatin was shown to induce thyroid neoplasms and forestomach papillomas [13], lovastatin induced liver tumours [14], and administration of simvastatin led to follicular cell adenomas [15]. In studies demonstrating a procarcinogenic effect, the doses of statins used were far higher than the biologically relevant concentrations used to treat hypercholesterolaemia in humans, possibly explaining these detrimental effects. Although some animal models have demonstrated anticarcinogenic effects of statins [16], a review of all available rodent carcinogenicity data for lipid-lowering agents concluded that, on balance, both statins and fibrates promoted cancer in rodents even at the equivalent concentrations prescribed in humans [17].

## Statins and the Prevention of Cancer—Observational Studies

A number of observational studies have supported the hypothesis of a protective effect of statins in terms of cancer risk [18–24]. One of the largest of these evaluated approximately 300,000 patients, using the Danish Cancer Registry, and found that any statin use reduced cancer incidence with an adjusted rate ratio (RR) for cancer overall of 0.86 (95% confidence interval (CI) 0.78 to 0.95) [25].

However, a number of other meta-analyses of observational studies of individual cancers have both disputed and supported a role for statins in the protection against cancer in equal measure [26–35]. One large meta-analysis including 25 observational studies and 17 randomised studies reviewed the effect of statins on all cancers, finding no effect on overall incidence (relative risk (RR) 0.96, 95% CI 0.72–1.2) [26]. However, a meta-analysis of 20 case-control studies suggested a significant beneficial association between statin use and risk of any cancer [35]. Although both large studies, these discrepancies reflect the inherent potential for bias, confounding, and heterogeneity between observational studies which could provide alternative explanations for any associations seen. An umbrella review and re-analysis of data from 43 meta-analyses, performed to determine the strength of the evidence available, found a statistically significant effect of statins on reducing cancer incidence in 10 types of cancer when using traditional methods; however, when the evidence was graded, accounting for between-study heterogeneity and potential bias, it was not found to be “convincing” for any cancer type [36].

## Statins and the Prevention of Cancer—Randomised Studies

A significant proportion of the evidence base underpinning the evaluation of aspirin as an anticancer drug originated from the long-term follow-up of large RCTs primarily designed to assess aspirin’s cardiovascular benefits [5, 37]. However, in similar randomised vascular trials of statin use, no difference has been shown in the incidence and risk of cancer. In the largest of these, the Heart Protection Study, 20,000 patients were randomised to 40 mg of simvastatin or placebo, with a reduction in cholesterol and proportional decrease in major vascular events seen in the statin cohort [38]. However, after a mean 11-year follow-up of survivors (*n* = 17,519), there was little evidence of a difference in the incidence of a first diagnosis of any cancer, either in-trial (first 5 years) or post-trial (subsequent 6 years) [39], or no difference by cholesterol level or tumour subtype.

In the early 2000s, a number of other RCTs assessing the effect of statins on the primary and secondary prevention of CVD consistently demonstrated vascular benefits. However, a large meta-analysis of 26 RCTs including 86,936 participants with 6662 incident cancers and 2407 cancer deaths showed that statins had a neutral effect on both incidence of cancer and cancer deaths, regardless of cancer subtype [40, 41]. To address specific concerns regarding the effect of lowering low-density lipoprotein (LDL) cholesterol with statins and potential risk of cancer, the Cholesterol Treatment Trialists’ (CTT) Collaboration performed a series of meta-analyses of individual patient data (*n* = 174,149) from 27 randomised vascular trials. They demonstrated that reducing LDL concentration with statins had no effect on either cancer incidence (RR per 1.0 mmol/L LDL reduction 1.00, 95% CI 0.96–1.04) or mortality (RR 0.99, 95% CI 0.93–1.06) [3, 42–44]. Statins were used for a median of 5 years, and no difference emerged regardless of whether trials were of statins versus control or trials of a more versus less intensive statin regime. Analyses of the effect of statins on individual cancers (23 different primary sites), by gender, older age, or statin type, were also consistent for a lack of association [45•]. These meta-analyses are probably the strongest available evidence to suggest that statins are unlikely to reduce the risk of incident cancer overall, at least in the short to medium term.

The implications for cancer-related mortality may be less clear. The included trials were for the primary and secondary prevention of CVD and the majority of patients enrolled would not be expected to have a cancer diagnosis at the time of randomization. Indeed, only cancers diagnosed after randomization were considered (1.4% developed cancer per year after randomization in the CTT study [46]), and it is not clear how many and for how long patients received the study drug after diagnosis. Whilst detailed information on statin use after a cancer diagnosis is not available in this data, statins are not routinely discontinued during cancer therapy, and therefore, some effect on mortality might be expected if there was a therapeutic role. Detailed follow-up of the data would be useful to make a more informed inference about the effect of allocation to statins on mortality outcomes in patients with cancer from these meta-analyses.

## The Role of Pharmaco-epidemiological Research

In contrast to the usual pathway for new drug development (phase I, II, and III trials), the rationale for a trial of a repurposed drug in the setting of cancer prevention or treatment can be informed by pharmaco-epidemiological research. In the past decade, there has been a marked proliferation in such research, due to a growing availability of population-based datasets drawn from a number of routinely collected health data sources (including electronic health records, pharmacy records, disease registries and outcome datasets) [47, 48]. Such research can leverage the availability of accurate prescription data in defined cohorts, with comprehensive data on relevant covariates and outcomes, to estimate the real-world effectiveness of statins in cancer prevention and treatment. There are notable examples where pharmaco-epidemiology has critically informed the justification for similar trials of other repurposed drugs in cancer prevention and treatment [8, 49]. Indeed, the AspECT trial, a 2 × 2 phase III trial of esomeprazole and aspirin for the prevention of a composite outcome of death, adenocarcinoma, and high-grade dysplasia in patients with Barrett’s oesophagus, was justified in part on the available pharmaco-epidemiological evidence and, ultimately, yielded some evidence for a chemo-protective effect consistent with this research [49]. However, there are also well-known examples where observational studies conflict with randomised data [50]. A common explanation for such discrepancies is the propensity for observational research to be susceptible to residual confounding, due to an imbalance of prognostic factors between treatment groups not accounted for in analyses, brought about by a lack of random treatment assignment [51]. However, observational studies can differ from randomised trials in other important ways to explain such discrepancies, including their design and analysis, with residual confounding a lesser concern in some instances [52]. To minimise these flaws, observational study design requires the emulation of a pragmatic hypothetical target trial (a randomised trial that would answer the question of interest), including explicit description of the causal framework, specifying the protocol of the target trial, robust reporting of study design, and a structured process for the analysis of study limitation [47, 53]. Use of such an approach in large healthcare datasets provides the opportunity to assess the association between a treatment and outcome using real-world data and helps to reduce the discrepancies between the effect estimates from observational studies and randomised trials [54••]. These are welcome steps towards improving the application of results from observational research, including, in this context, to improve their utility in informing the justification for or against a trial of adjuvant statin therapy.

An analysis of the electronic health records of 733,804 UK adults, explicitly emulating a target trial of statins and cancer, found little evidence to indicate that statin therapy influences cancer incidence after 10-year follow-up [54••], regardless of cancer subtype, and consistent with findings of the CTT Collaboration. The authors further demonstrated that the failure in replicating observational study results arose from the deviation from the basic principles of a trial design [54••]. Eligibility criteria included LDL cholesterol level < 5 mmol L^−1^,which may have introduced some confounding, channelling people with diabetes into the statin group (diabetes being an indication for statin therapy irrespective of LDL), and diabetes is a known risk factor for cancer. However, pharmaco-epidemiological research could be utilised much like this example to supplement the evidence base for a phase III repurposed drug trial, but perhaps also to provide more timely results to support clinical decision making.

## The Role of Mendelian randomization

Conventional observational epidemiological studies are vulnerable to various biases (e.g., residual confounding, reverse causation, and measurement error) which undermine causal inference. Mendelian randomization (MR) uses genetic variants to proxy risk factors to generate more reliable evidence in support of causal effects of these factors on health outcomes in observational settings [9]. Since germline genetic variants are randomly assorted at meiosis and fixed at conception, MR analyses should be less prone to confounding than conventional observational studies (e.g., by indication, lifestyle factors) and are not subject to reverse causation bias. Further, measurement error in genetic studies is often low and MR estimates the life-long effects of risk factors on health outcomes, allowing sufficient time for diseases with long latency periods—like cancer—to develop an advantage over secondary analyses of short-term RCTs. MR relies on sound knowledge of the mechanism of action and existence of a relevant genetic variant, and therefore, it may not be possible or useful for all drug evaluations. However, findings from well-conducted and adequately powered MR studies, with appropriate sensitivity analyses to evaluate the assumptions of MR [55], could provide a useful contribution to the evidence synthesis of statins as a potential preventive agent or therapeutic treatment for cancer.

A commonly employed approach for inferring causal effects of statins on cancer in an MR context is to utilise genetic variants in the gene encoding the drug target of statins, *HMGCR*, that are robustly associated with LDL cholesterol levels as proxies for pharmacological inhibition of this drug target. One such study employed the LDL cholesterol-lowering T-allele of rs12916 (located within *HMGCR*) as a proxy for HMG-CoA reductase inhibition to examine the effects of its inhibition on risk of cancer in the Malmö Diet and Cancer Study [56]. The authors reported little evidence for an effect of HMGCR inhibition on overall cancer risk (HR equivalent to 0.07 mmol/L lowering of LDL cholesterol 0.99, 95% CI 0.95–1.02), though statistical power was modest for this analysis (*n* = 6528 cases). In an analysis of 22,773 men with prostate cancer and 23,050 controls in the Prostate Cancer Association Group to Investigate Cancer Associated Alterations in the Genome (PRACTICAL) consortium, a weak protective effect was reported of the rs12916-T variant with prostate cancer risk (OR 0.97, 95% CI 0.94–1.00; *p* = 0.03) [57], and little evidence apparent of an association with stage or tumour grade. Three variants in *HMGCR* were evaluated in relation to risk of breast cancer in 122,977 cases and 105,974 controls in the Breast Cancer Association Consortium (BCAC) [58]. Similarly weak evidence was demonstrated for a protective effect of HMGCR inhibition on cancer risk (OR equivalent to 1 mmol/L lowering of LDL cholesterol 0.86, 95% CI 0.73–1.02, *p* = 0.09) [58], with comparable odds when analyses were restricted to status of oestrogen receptor. Most recently, a study of genetically proxied HMGCR inhibition, equivalent to a 1-mmol/L reduction in LDL cholesterol, demonstrated robust evidence of a lower risk of epithelial ovarian cancer (OR 0.60, 95% CI 0.43–0.83; *p* = 0.002) in both the general population and in BRCA 1/2 mutation carriers (HR 0.69, 95% CI 0.51–0.93; *p* = 0.01) [59••], consistent with pre-clinical and epidemiological studies, perhaps warranting further investigation in this high-risk group. Going forward, the integration of MR with other complementary genetic approaches may provide a promising opportunity for validating the effects of drug targets like HMGCR on cancer outcomes. For example, Zheng et al. have reported that combining evidence from MR and colocalization analysis, a technique that helps to distinguish shared causal variants across traits from genetic confounding due to linkage disequilibrium, can substantially increase the likelihood of predicting positive clinical trial outcomes leading to regulatory approval of new drugs [60].

Extension of the MR approach to various other cancers within the context of adequately powered datasets along with the application of this approach to the study of cancer progression or recurrence [61], rather than cancer incidence, remains a fruitful area of investigation. The latter is important as the utility of a particular agent in the context of risk reduction (primary prevention) may not necessarily translate to a therapeutic effect in the context of disease progression (tertiary prevention), and vice versa. Insights gained from the use of MR in interrogating causal relationships between statin use, cancer occurrence, and cancer outcomes can therefore provide complementary evidence to other study designs on the potential role of long-term statin therapy in reducing incidence or progression of cancer.

Table 1 summarises the types of evidence evaluating statin therapy in the prevention of cancer.

**Table 1:** A summary of the different type’s evidence for statins in the chemoprevention of cancer, with examples

Type of evidence	Study	Number of studies/cases	Results (95% confidence interval)
In vivo evidence at biologically relevant doses	Newman, JAMA [17] (1996)	Review of rodent carcinogenicity studies	Statins increase incidence of cancer
Statins and cancer prevention			
Meta-analyses of epidemiological data	Kuoppala, Eur J Cancer [26] (2008) Taylor, Eur J Cancer Prevention [35] (2008)	42 differing study designs *n* = 67,432 cases 20 case-control *n* = 100,129	RR 0.96 (0.72–1.12) OR 0.71 (0.56–0.89)
Meta-analyses of RCTs	Dale, JAMA [40] (2006)	26 RCT *n* = 6662 cases	OR 1.02 (0.97–1.07)
	Kim, Indian J Cancer [41] (2017)	21 RCT *n* = 32,615 on statin	RR 0.97 (0.92–1.02)
Individual patient data from RCTs	CTT, Lancet [42] (2012), PLOS [46] (2012), Lancet [44] (2010), Lancet [45•] (2019)	27 RCT *n* = 175,000	Incidence RR 1.00 (0.96–1.05) Mortality RR 1.00 (0.93–1.08)
Pharmaco-epidemiology using a target trial design	Dickerman, Nature Medicine [54••] (2019)	*n* = 28,408 cases	Cancer-free survival difference − 0.5% (−1.0%–0.0%)
Mendelian randomization	Orho-Melander, Int J Epidemiol [55] (2018)	*n* = 6528	HR equivalent to 0.07 mmol/L LDL lowering 0.99 (0.95–1.02)
	Bull, Cancer Medicine [56] (2016)	*n* = 22,773 prostate	OR 0.97 (0.94–1.00)
	Nowak, Nature communications [57] (2018)	*n* = 122,977 breast	OR equivalent to 1 mmol/L LDL lowering 0.86 (0.73–1.02)
	Yarmolinsky, JAMA [58] (2020)	*n* = 25,509 ovarian *n* = 3887 BRCA 1/2	OR 0.6 (0.43–0.83) OR 0.69 (0.51–0.93)

## Statins and the Adjuvant Treatment of Cancer

### Evaluating “Traditional” Evidence for Statins in the Treatment of Cancer

A possible role for statins in the adjuvant setting of cancer has been suggested by a recent in vitro study, where statins were shown to reduce the outgrowth of metastases in two breast cancer cell lines [62]. It has therefore been suggested that statins could delay breast cancer recurrence and reduce mortality [63]. A number of population-based cohort studies have demonstrated that statins may reduce cancer-specific mortality in breast, colorectal, and lung cancer [64–66]. One of the largest of these reviewed statin use in Danish residents diagnosed with stage I–III invasive breast cancer (*n* = 18,769). Simvastatin users developed approximately 10 fewer breast cancer recurrences per 100 women after 10 years, with 10-year adjusted HR 0.70 (95% CI 0.57–0.86) [67, 68]. In a meta-analysis of 10 observational studies, including 32,373 patients with breast cancer, lipophilic statin use was shown to be associated with improved recurrence-free survival (HR 0.64, 95% CI 0.53–0.79) at 5-year follow-up [69]. However, many of these studies are affected by collider stratification bias—unmeasured confounding induced by selected bias—and an evaluation of alternative explanations is crucial [70].

The potential survival benefit of statins for patients with malignancy has been further evaluated in a meta-analysis of 95 cohort studies, including 1,111,407 individuals. Subgroup analyses according to initiation of statins showed that post-diagnosis statin use was associated with a significant improvement in recurrence-free survival (HR 0.65; 95% CI 0.54–0.79), which was less convincing for pre-diagnosis use (HR 0.86, 95% CI 0.77–0.96); a similar association was seen for overall survival (OS) [71]. Most studies identified were in prostate, breast, and colon cancer, with consistent benefits seen across these three tumour types. However, a recent umbrella review of meta-analyses, an appraisal of high-level evidence, evaluating data from 112 meta-analyses of observational studies and 144 meta-analyses of RCTs, found only class III (suggestive) evidence of decreased cancer-related mortality in patients with cancer who had statins post-diagnosis, concluding limited convincing evidence of an effect of statins on cancer mortality in the adjuvant setting [72].

The evidence for the use of statins post diagnosis of cancer appears most abundant in the setting of breast cancer [69, 73, 74]. However, as with other tumour types, most data exists in the observational setting, with little randomised evidence. The Breast International Group’s large RCT (BIG 1-98) appears to be the only observational study of statin use in the adjuvant setting of breast cancer in the context of a randomised controlled trial of endocrine therapy. Survival of 8010 postmenopausal women with early-stage, hormone receptor–positive breast cancer was assessed, in whom 789 initiated cholesterol-lowering medication (CLM) during endocrine therapy [75]. In patients commenced on CLM (including non-statins), an association with improved disease-free survival (HR 0.79; 95% CI 0.66 to 0.95), breast cancer–free interval (HR 0.76; 95% CI 0.60 to 0.97), and distant recurrence–free interval (HR 0.74; 95% CI 0.56 to 0.97) was demonstrated [75].

### Pharmaco-epidemiological Research in the Adjuvant Setting of Cancer

The majority of observational research examining the association between statin use and survival after a diagnosis of cancer compares outcomes between current users (including prevalent users) and never users. This answers a fundamentally different question to that posed by RCTs: whether allocation to statins (new users) compared with no statins at the point of randomization affects survival. There have been considerable methodological advancements to improve consistency of estimates from observational research with trials, including *new-user designs* (which restricts analysis to individuals under observation at the start of the treatment of interest) [76] and their incorporation into a wider paradigm shift: emulation of a target trial using observational datasets [53]. The latter requires the explicit alignment of the protocol for an observational study to that of the hypothetical “target” trial (described previously), specifically with regard to the eligibility criteria, treatment strategies, assignment procedures, outcome, causal contrasts of interest (intention to treat and per-protocol effects), and analysis plan [53]. To date, a single study has employed such an observational analogue of a target trial in this context—a retrospective analysis of US SEER (Surveillance, Epidemiology, and End Results) data from 17,372 patients. This study found that initiation of statins within 6 months after cancer diagnosis (stages I–III colorectal, breast, prostate, and bladder) did not improve 3-year cancer-specific or overall survival [77]. This approach accounted for potential selection and immortal time bias, and the effect of residual confounding was likely to be negligible (HR for OS 1.00, 95% CI 0.88–1.15), although longer-term follow-up is important. The application of this methodology to other cancer types would be welcome in informing the decision to proceed to a trial.

### Evidence from Mendelian randomization

There is now a large body of evidence using MR to evaluate the effect of genetic risk on cancer incidence, with a number specifically evaluating HMGCR inhibition. However, few studies have attempted to identify variants associated with cancer progression, and those that have are mostly small [61, 78]. A number of issues have been identified in Mendelian randomization of progression^61^. Compared to cancer incidence, which is usually a binary outcome, disease progression is more complex to measure and harder to define. There is an increased potential for confounding through collider bias, leading to a possible spurious association between the genetic variant and progression. Furthermore, MR requires availability of large data sets with detailed follow-up of both progression and genetic data [61]. Therefore, for a MR study of progression to be successful, large, and likely collaborative, RCTs collecting DNA as a standard are required. To date, few studies have used MR to identify factors influencing disease progression [78–80], and to our knowledge, none evaluating HMGCR inhibition as a proxy for statin therapy.

## Randomised Trials Investigating Statins in Cancer

Most randomised trials investigating statins in cancer have been in advanced cancers, with few in the adjuvant setting, and most-earlier phase (non-confirmatory) studies with relatively small sample sizes (Table 2).

**Table 2:** Randomised trials investigating statins in patients with cancer

Study	Phase	Cancer	Previous treatment	Statin	Standard treatment	No. of patients	Median OS (months) and HR statin vs placebo
Kawata 2001, BJC [81]	II	Advanced liver	No	Pravastatin 40 mg	TAE, F/u	83	18 vs 9 m HR 0.50, *p* = 0.006
Garwood 2010, BCRT [82]	II	DCIS or stage I breast	No	Fluvastatin 20 vs 80 mg	Statin 3–6 weeks before surgery	40	Ki67 reduced, CC3 increased
Kim 2014, EJC [83]	III	Advanced gastric	No	Simvastatin 40 mg	Capecitabine and cisplatin	244	11.6 vs 11.5 m, HR 0.97, *p* = 0.82
Hong 2014, CCP [84]	II	Advanced pancreas	No	Simvastatin 40 mg	Gemcitabine	114	6.6 vs 8.9 m, *p* = 0.83
Lim 2015, BJC [85]	III	Metastatic CRC	Yes	Simvastatin 40 mg	XELIR/FOLFIRI	269	15.3 vs 19.2 m, *p* = 0.83
Konings 2010, EJC [86]	II	Unresectable gastric	No	Pravastatin 40 mg	Epirubicin, Cisplatin, Cape	30	8 vs 6 m, *p* > 0.05
Seckl 2017, JCO [87]	III	SCLC	No	Pravastatin 40 mg	Etoposide and Cis/Carbo	846	14.6 vs 14.6 m, HR 1.01, *p* = 0.90
Alexandre 2017, BMJ Gut [88]	Feasibility	Resectable oesophageal	No	Simvastatin 40 mg	Surgery	32	HR 1.56, *p* = 0.716*
Jouve 2019, J Hepatol [89]	III	Advanced liver	No	Pravastatin 40 mg	Sorafenib	323	10.7 vs 10.5, HR 1.00, *p* = 0.975

*Feasibility study, not powered for overall survival analysis

Any favourable effects of statins on cancer-related mortality apparent from epidemiological data have not always been corroborated by clinical trials, and the trial evidence demonstrated in Table 2 has not all been sufficiently robust to confirm or refute the effectiveness of statins in cancer. In Kawata et al.’s phase II study of advanced hepatocellular cancer, patients received treatment with transcatheter arterial embolisation and 5-fluorouracil chemotherapy and were randomised to 40 mg of pravastatin or control, with a doubling of median OS seen from 9 to 18 months (*p* = 0.006) [81]. This study had a sample size of only 83 patients; and the findings were subsequently not replicated in the PRODIGE-11 trial, an RCT of 323 patients with advanced hepatocellular carcinoma randomised to first line sorafenib-pravastatin (40 mg) combination therapy or sorafenib alone, which demonstrated no significant difference in OS between treatment arms [89]. The justification to proceed with a phase III multicentre trial in this context was based on more traditional evidence from pre-clinical and observational studies, one meta-analyses and an initial phase II study, with no contribution to the evidence base from more contemporary methods.

A number of other trials are currently underway; however, the use of more contemporary methods to support the go/no-go decision in proceeding with a clinical trial may help to reduce the likelihood of negative trials.

## The Tolerability of Statin Therapy

The model for evaluating repurposed drugs should consider its toxicity and tolerability, particularly in the adjuvant setting, where significant or chronic toxicity may limit adherence. A well-documented adverse effect of statin therapy is muscle toxicity, including myopathy and rhabdomyolysis. Myopathy occurs in fewer than 1 in 10,000 individuals, although risk is considered related to both statin type and dose [90]. Persistent and asymptomatic increases in serum transaminases have been reported, although usually recover on discontinuation. There have however been rare post-marketing reports of hepatic failure. Generally, statins are considered well tolerated, and reasonably good 5-year compliance has been demonstrated in prior RCTs [38]. However, meta-analyses of individual patient data evaluating the safety of patients enrolled onto RCTs of statins in cardiovascular disease would provide a more comprehensive summary of the risk of adverse events. The CTT Collaboration is currently examining this data [91] and evaluation of safety would be an important part of any new phase III cancer trial.

## A Potential Model for Evaluating Repurposed Drugs in Cancer Chemoprevention

Figure 1a demonstrates the conventional pathway of drug development. Following the discovery of a promising compound, supporting evidence from pre-clinical and observational data typically leads to clinical trials evaluating safety and efficacy. On average, the time between drug discovery and clinical trial is 9 years, with a huge cost involved, and success rate of less than 10% [92, 93]. Compared to the traditional drug discovery process, drug repurposing can considerably reduce the cost and time to bring a new treatment to patients, with many of the early steps in the drug development pathway already completed. The main justification for reusing licenced drugs for a new indication is that they have known mechanisms of actions and toxicity profiles [94]. However, despite these major advantages of drug repurposing, the issue of efficacy remains the same [95], with overall success rate shown to be less than 6%, much like new oncological drug discovery [92, 96]. Therefore, careful consideration is required before embarking on a clinical trial [94]. The well-established model for drug discovery (Fig. 1a) is not designed for drug repurposing [97]. Major efforts need to be made to improve the rate of efficacy, perhaps considering a new adapted model for evaluating repurposed drugs. Figure 1b proposes a new evidence framework, particular to repurposed drugs, suggesting concurrent appraisal of already available data and the review of further evidence, prior to conducting a large RCT.

## Conclusion

In conclusion, whilst most pre-clinical and observational evidence appears to support the use of statins in the prevention of cancer, results from the randomised vascular setting, pharmaco-epidemiological research using a target trial design, and evidence from MR have all been less convincing in most tumour types. There is little supportive evidence of the beneficial effects of statins as a treatment for cancer from phase III trials; however, most inferences have been limited to the non-curative setting. The challenge is, therefore, to weigh up the evidence from all sources as systematically and fairly as possible, as proposed by our model for evaluating repurposed drugs (Fig. 1a, b), to decide whether a trial in the adjuvant setting is justified on a tumour site-by-site basis. Further evaluation of the role of statins in the adjuvant setting may be justified, with breast cancer being one such candidate disease site to potentially investigate based on the evidence presented here. Future observational research emulating a hypothetical RCT, “a target trial”, in the adjuvant setting, and use of Mendelian randomization may help inform the decision to proceed to a potential phase III RCT of a repurposed drug.

## Abbreviations

CI: Confidence interval
CLM: Cholesterol-lowering medication (CLM)
CTT: Cholesterol Treatment Trialists
CVD: Cardiovascular disease
HMGCR: 3-Hydroxy-3-methylglutaryl-coenzyme A reductase
HR: Hazard ratio
LDL: Low-density lipoprotein
MR: Mendelian randomization
OR: Odds ratio
RR: Relative risk
RCT: Randomised controlled trial
